# Uncovering Pathways to Occupational Health and Safety (OHS): Presenteeism and Psychosocial Safety Climate in the JD–R–OHS Model

**DOI:** 10.1002/smi.70222

**Published:** 2026-09-15

**Authors:** Jafar Akbari, Paula Brough, Xi Wen (Carys) Chan

**Affiliations:** ^1^ Centre for Work, Organisation and Wellbeing Griffith University Brisbane Australia; ^2^ Department of Management Griffith Business School Griffith University Brisbane Australia

**Keywords:** health‐related work functioning, job demands resource model, presenteeism, psychosocial safety climate, safety leadership, safety outcomes

## Abstract

Occupational health and safety (OHS) remains a critical concern, yet the psychosocial mechanisms linking job demands and leadership to safety behaviors and motivation remain underexplored within the Job Demands–Resources (JD‐R) framework. This three‐wave longitudinal study extended the JD‐R‐OHS model to examine Psychosocial Safety Climate (PSC) and health‐related work functioning, operationalized as presenteeism using the Stanford Presenteeism Scale (SPS‐6), in relation to safety outcomes. Structural equation modeling distinguished pathways linking safety‐specific leadership and job‐family demands with safety compliance, participation, and motivation. Findings provided mixed support for the proposed model. PSC was the most consistently supported organisational mechanism, particularly linking safety‐specific leadership with safety participation and motivation. In contrast, pathways involving perceived job and family demands and health‐related work functioning received limited support. These findings suggest that organisational climate resources may be more influential than individual health‐related work functioning in explaining safety outcomes and highlight PSC as a robust mechanism warranting investigation.

## 1 Introduction

OHS is a fundamental aspect of human resource management (HRM), focussing on the systematic identification, assessment, and management of workplace hazards that jeopardize employee health and well‐being (R. Liu et al. 2023). According to the International Labor Organization (ILO 2024), work‐related factors contribute to approximately 2.93 million fatalities and 395 million non‐fatal injuries globally each year. The economic burden of workplace incidents warrants significant attention, with the implementation of health and safety strategies estimated to yield global savings of approximately USD 361 billion (ILO 2024). Therefore, the development of a resource‐based mechanism and an integrated approach to identify the factors influencing safety behaviors is critical for HRM. This necessity is underscored by the legal and ethical obligations of employers to ensure the OHS of their employees (Gannon and Paraskevas 2020).

Exposure to work‐related psychosocial hazards is increasingly recognised as a significant threat to occupational health and safety, with considerable implications for employees, organisations, and the broader economy (Cobb 2022; Lindholm et al. 2020). Effective management of these hazards enables organisations to allocate resources toward enhancing employee learning opportunities and safety practices (Naji et al. 2021), thereby fostering a positive psychosocial climate that is correlated with increased safety compliance and participatory behaviors. These behaviors are associated with reduced adverse safety outcomes, including injuries and accidents (Sinclair et al. 2010; Vinodkumar and Bhasi 2010). The JD‐R model, developed by Bakker and Demerouti (2007), offers a robust framework within occupational health psychology to investigate the effects of job demands on employee motivation and compliance with OHS requirements. This model posits that while job demands may deplete motivation, job resources can replenish it, elucidating pathways for safety behaviors and health impairment (Seo et al. 2025).

Recent developments have expanded the JD–R model beyond its original focus on employee well‐being and work engagement to encompass broader occupational health and safety outcomes. Contemporary research increasingly conceptualizes safety‐related behaviors (Margheritti et al. 2023), safety motivation, and psychosocial risk management (W. Liu et al. 2025) as outcomes stemming from both motivational and health impairment processes. These developments support the use of the JD–R framework for examining psychosocial risk management and organisational safety processes, particularly where climate‐level resources such as psychosocial safety climate (PSC) are expected to shape both motivational and health‐related pathways (Tummers and Bakker 2021; Mazzetti et al. 2023; Seo et al. 2025).

This study employs the JD‐R framework to explore the influence of job demands and resources on safety outcomes. A significant proportion of workplace incidents can be attributed to unsafe behaviors (Difford 2011; Gantt 2017). Consequently, it is essential to recognize that motivation to comply with OHS standards is critical for reducing or eliminating accidents (Bowdler et al. 2023; Hedlund et al. 2016). Recent developments have extended the application of the JD‐R model beyond employee well‐being and work engagement to encompass safety‐related outcomes, highlighting the importance of integrating organisational resources, leadership, and psychosocial work environments in explaining employee safety behaviors (Bakker et al. 2023; Lesener et al. 2019). Accordingly, the present study proposes a JD‐R‐OHS framework to examine the influence of perceived job demands (PJD), perceived family demands (PFD), and resources on employee health and safety, as well as the implications for compliance with workplace safety protocols. Although previous studies have examined job demands and resources, PSC, safety‐specific leadership, and presenteeism independently, comparatively few have integrated these constructs within a single longitudinal framework. In particular, limited research has examined whether PSC serves as an organisational mechanism linking safety‐specific leadership and psychosocial job demands with safety motivation and safety behaviors, while accounting for the conditional role of presenteeism. Addressing these gaps contributes to the growing integration of occupational health psychology and organisational safety research and extends contemporary applications of the JD‐R framework within OHS contexts. Therefore, this study expands the JD‐R‐OHS framework by investigating the contributions of organisational climate and health‐related work functioning to safety outcomes over time. Specifically, PSC is proposed to act as an upstream organisational resource, while presenteeism is included as an additional health‐related work functioning variable to assess its potential explanatory power for safety outcomes. Given the limited and inconsistent evidence linking presenteeism to safety‐related processes, its role in this model is considered exploratory rather than central.

In this study, presenteeism is operationalized as health‐related work functioning using the Stanford Presenteeism Scale (SPS‐6); therefore, throughout the empirical analyses, the term presenteeism refers specifically to SPS‐6‐assessed health‐related work functioning. Higher total SPS‐6 scores indicate better health‐related work functioning, reflecting a greater ability to concentrate and complete work despite health problems, rather than greater sickness attendance, attendance pressure, or productivity loss.

### 1.1 Theoretical Framework and Hypotheses Development

#### 1.1.1 Conceptual Framework of the JD‐R‐OHS Model

Job demands (JD), the physical, psychological, social, and organisational aspects of work requiring sustained effort, are associated with physiological and psychological costs, reflecting the burden placed on employees in both their physical and mental health (Demerouti et al. 2001). Research consistently links JD to detrimental health and performance outcomes; however, family demands (FD) have been comparatively understudied despite their significant influence on employee well‐being (Ertel et al. 2008; Ji et al. 2023) and job performance through the mechanism of home‐work interference (Demerouti et al. 2010). Moreover, evidence also shows that family resources, particularly family support, shape the effects of JD on outcomes such as turnover intentions, highlighting the importance of FD management (Le et al. 2023). Guided by the JD‐R health‐impairment pathway, where JD and FD erode employee resources and impair outcomes, this study hypothesizes the following:

H 1a PJD and PFD will be negatively associated with health‐related work functioning (1) and perceptions of PSC (2).

H 2a PJD and PFD will be negatively associated with safety outcomes (Safety Compliance (SC), Safety Participation (SP), and Safety Motivation (SM)).

Job resources (JR), including support from management and leadership, counteract the negative effects of job demands and facilitate employee functioning (Krick et al. 2021; Richter et al. 2021). Supportive leaders empower employees (Delizonna 2017; Islam et al. 2024), strengthen trust and belonging (Kang et al. 2023), shape organisational climate (Coronado‐Maldonado and Benítez‐Márquez 2023), and enhance team performance (Sethibe 2018). In the OHS domain, leadership is fundamental to cultivating safety culture, promoting adherence to best practices (Basahel 2021; Nasim et al. 2022) and communicating safety priorities (J. Mullen et al. 2017).

Studies indicate that safety leadership (SL) styles are more effective than general leadership styles in fostering safety practices within organisations. Leaders who embody a safety‐specific transformational leadership (SSTL) approach communicate a shared vision of safety, empowering employees to utilize their energy, skills, and self‐efficacy to achieve this vision (Barling et al. 2002; Inness et al. 2010). This leadership style is a more reliable predictor of safety behaviors compared to general transformational leadership (de Koster et al. 2011; J. Mullen et al. 2017). Additionally, the tactics inherent in SSTL promote safety and enhance performance (Lu et al. 2019). Despite the scholarly focus on SSTL, many employees primarily encounter passive leadership styles (Aasland et al. 2010). Research indicates leaders can demonstrate both transformational and passive styles as viable options (J. Mullen et al. 2011). Individuals exhibiting a safety‐specific passive leadership (SSPL) style tend to show limited responsiveness to workers' safety and health needs, often reacting only when a safety‐related incident is imminent or has already occurred (Smith et al. 2016). Recent studies indicate the SSPL style may adversely affect worker health (Diebig and Bormann 2020) and safety outcomes (Arief et al. 2020). Recent evidence further suggests that passive (laissez‐faire) leadership should not be viewed merely as the absence of effective leadership but rather as a leadership style with distinct implications for occupational safety. Passive leaders often fail to communicate safety expectations, provide timely feedback, or intervene when hazards arise, contributing to weaker safety climates, lower safety motivation, and poorer safety behaviors (Ta et al. 2022; Lyubykh et al. 2022). Despite growing evidence regarding these relationships, the organisational mechanisms through which passive leadership operates, particularly via PSC within the JD–R framework, remain comparatively underexplored. Given the significant roles of SSTL and SSPL, as well as the limited studies examining SSPL's impact on OHS outcomes, this study proposes the following hypotheses:

H 1b SSTL will be positively associated with health‐related work functioning (1) and PSC (2), whereas SSPL will be negatively associated with health‐related work functioning (3) and PSC (4) within organisational settings.

H 2b SSTL will be positively associated with safety outcomes (1), including SC, SP, and SM, whereas SSPL will be negatively associated with these outcomes (2) among employees.

In the JD‐R‐OHS model of this study, presenteeism (operationalized as health‐related work functioning) and PSC are positioned as mediators and moderators within the health impairment and motivational pathways, respectively. The study analyses these variables and examines three key safety outcomes: SC, SP, and SM. Hypotheses regarding the direct effects of presenteeism and PSC on these outcomes are proposed below:

H 3a Higher health‐related work functioning will be positively associated with safety compliance, safety participation, and safety motivation.

H 3b Higher PSC will be positively associated with safety compliance, safety participation, and safety motivation.

SC and SP represent distinct dimensions of safety behavior that together comprise safety performance. SC entails following established safety procedures and utilizing protective equipment to uphold a safe working environment. In contrast, SP includes discretionary behaviors that foster the safety culture, such as aiding coworkers and engaging in safety initiatives (Neal et al. 2000). Employees are expected to enhance their safety behaviors through compliance with safety policies, illustrating a reciprocal relationship (Syed‐Yahya et al. 2022). Though interrelated, these constructs predict safety outcomes, including workplace accidents and injuries (X. Yang et al. 2021). Moreover, organisations prioritizing safety significantly enhance compliance and participation by embedding these aspects within their culture (Zohar and Luria 2010).

SM reflects an individual's willingness to engage in safety behaviors and the perceived value of these practices. Individuals are more motivated to adhere to safe work practices and participate in safety initiatives when they experience a positive safety climate (Neal and Griffin 2006). A robust SM is critical for mitigating accidents and sustaining safety levels by applying scientific principles and established procedures. Effective SM enhances compliance with safety protocols, protecting both workers' well‐being and organisational integrity (Ying et al. 2012). While previous research identifies SM as a potential mediator or moderator within the JD‐R framework, this study focuses on SM as a primary safety outcome due to its pivotal role in shaping the safety climate (Syed‐Yahya et al. 2022) and influencing safety behaviors. The objective is to analyze how SM is influenced by health impairment and motivational pathways.

#### 1.1.2 The Impairment Path of JD‐R‐OHS: Presenteeism and Safety Outcomes

Presenteeism is defined as the phenomenon wherein employees are physically present at their workplace but are unable to perform effectively due to illness or other medical conditions (Hemp 2004). It can be conceptualized as a mediating variable in the health impairment pathway, where sustained job demands compromise employees' physical and psychological resources (Demerouti et al. 2001). This phenomenon may lead to increased strain, stress, and elevated burnout levels, resulting in adverse health outcomes (Demerouti et al. 2009; Freeling et al. 2020). Although presenteeism is associated with various health issues, including burnout (McGregor et al. 2016), it also contributes to the worsening of physical and psychological conditions, which poses risks to employee health and organisational productivity (Li et al. 2022, 2019).

Recent conceptual and methodological developments have further clarified that presenteeism encompasses two related but distinct perspectives. One conceptualization defines presenteeism as sickness attendance (i.e., attending work despite illness), whereas the other focuses on productivity loss attributable to health problems while at work. Although both perspectives are informative, they represent different theoretical constructs and require different measurement approaches. The present study utilizes a health‐related work functioning framework, operationalizing presenteeism through the Stanford Presenteeism Scale (SPS‐6). The SPS‐6 evaluates employees' ability to concentrate, complete work tasks, and maintain overall functioning while experiencing health‐related issues. In line with the scoring method used in this study, higher total SPS‐6 scores indicate improved health‐related work functioning. Consequently, the empirical analyses conceptualize presenteeism as a measure of health‐related work functioning rather than as a determinant of sickness attendance or attendance‐related decisions.

Positioning health‐related work functioning within the health impairment pathway is justified by its association with job demands and the resulting negative outcomes. High job demands contribute to poorer health‐related work functioning and adversely affect employee health (Gustafsson et al. 2020). Studies have documented presenteeism as an occupational hazard associated with reduced safety awareness (Guo et al. 2023), diminished perceptions of the workplace safety climate (Kinman and Clements 2022), and an increased incidence of accidents (Ishimaru et al. 2019). Health‐related work functioning, operationalized as presenteeism via the SPS‐6, was incorporated into the current model as a measure of employees' functioning while managing health issues, rather than as a proxy for absenteeism. Although prior research indicates that health issues can impair workplace functioning, the evidence concerning the extent to which presenteeism predicts safety outcomes remains limited and inconsistent. Consequently, presenteeism was examined as a provisional mechanism within the impairment pathway, with the expectation that any associations with safety outcomes would necessitate cautious interpretation. Considering these identified gaps, this study posits the following hypotheses:

H 4a The negative associations between PJD and PFD and safety outcomes (SC, SP, and SM) (1) will be sequentially mediated by lower health‐related work functioning. The positive associations between SSTL and safety outcomes and the negative associations between SSPL and safety outcomes (2) will also be mediated by health‐related work functioning.

H 5a Health‐related work functioning will moderate the relationships between job demands and safety outcomes. Higher health‐related work functioning will attenuate the negative associations between PJD/PFD and safety compliance, safety participation, and safety motivation.

H 5b Health‐related work functioning will moderate the relationships between safety leadership and safety outcomes. Higher health‐related work functioning will strengthen the positive associations between SSTL and safety outcomes and attenuate the negative associations between SSPL and safety outcomes.

#### 1.1.3 The Motivational Pathway of JD‐R‐OHS: The Effects of PSC on Safety Outcomes

The motivational pathway is often examined through the JD‐R model. In this framework, Job Resources (JRs) that facilitate the attainment of work goals, alleviate job demands, or support personal growth contribute to increased work engagement and motivation, thus establishing a ‘motivational process’ (Bakker and Demerouti 2007; Demerouti et al. 2001). JRs initiate this pathway, influencing mediators and predicting significant outcomes. This study incorporates PSC into the JD‐R model to evaluate its effectiveness in enhancing safety outcomes. PSC reflects a dimension of organisational climate encompassing shared perceptions of policies and practices aimed at safeguarding workers' psychological health and safety (Dollard and Bakker 2010).

Recent PSC research has increasingly conceptualized the construct as an indicator of organisational psychosocial risk management rather than solely employees' perceptions of management commitment. Within the JD‐R framework, PSC serves as the organisational context through which leadership impacts employee motivation, safety behaviors, and psychological well‐being. Consequently, this study identifies PSC as the primary theoretical mechanism that connects safety‐specific leadership with occupational health and safety outcomes. Contemporary studies demonstrate that PSC is associated with lower psychosocial hazards, stronger employee well‐being, improved safety behaviors, and more effective organisational management of psychological health risks. These developments reinforce PSC as an upstream organisational resource within the JD–R framework and support its practical relevance for improving both employee well‐being and occupational safety outcomes (Mirza et al. 2022; W. Liu et al. 2025).

Organisations with high PSC prioritize workforce well‐being and safety (Peters et al. 2021). This study hypothesizes the factors linking JRs to PSC and their impact on safety outcomes. In this framework, two key stakeholders play a crucial role in establishing a robust PSC. Leaders allocate resources, develop policies, and engage employees in designing safe work environments, which are essential for enhancing well‐being and productivity (Dollard et al. 2019). Employees then translate these initiatives into observable behaviors. PSC shapes employees' perceptions, emotions, and actions, either facilitating or impeding individual and team functioning (Dollard and Bakker 2010). Research indicates that PSC buffers the effects of high job demands and JRs on health and safety outcomes (Peters et al. 2021). A growing body of research has explored the role of PSC; however, its significance and application within the JD‐R model, particularly in relation to health and safety outcomes, remain inadequately understood. This study aims to investigate PSC within the OHS motivational pathway, focussing on the interplay between leadership and workplace climate in relation to safety outcomes. The following hypotheses are proposed:

H 4b The negative associations between PJD and PFD and safety outcomes (1) will be mediated by PSC. The positive associations between SSTL and safety outcomes and the negative associations between SSPL and safety outcomes (2) will also be mediated by PSC.

H 6 PSC will moderate the relationships between (a) safety leadership styles (SSTL and SSPL) and safety outcomes (SC, SP, and SM), and (b) job demands (PJD and PFD) and safety outcomes, such that higher levels of PSC will attenuate the detrimental effects of job demands and SSPL on safety behavior and motivation, while amplifying the positive influence of SSTL on these safety outcomes.

**FIGURE 1:**
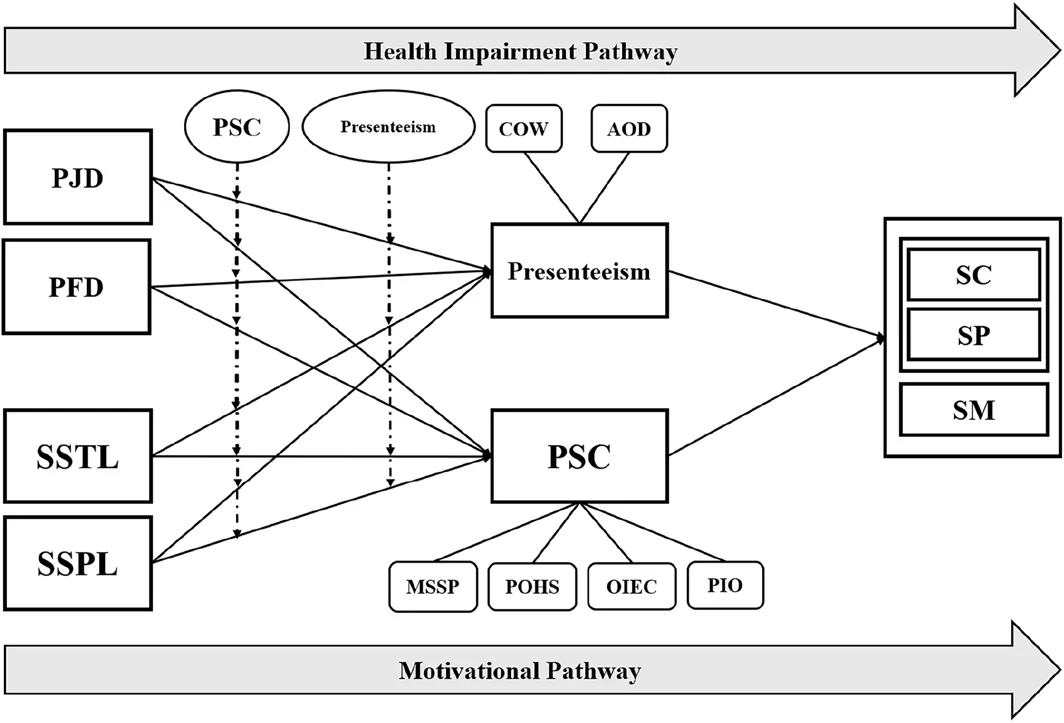
Research model for the study. PFD, Perceived Family Demands; PJD, Perceived Job Demands; PSC, Psychosocial Safety Climate; SC, Safety Compliance; SM, Safety Motivation; SP, Safety Participation; SSPL, Safety‐Specific Passive Leadership; SSTL, Safety‐Specific Transformational Leadership. In the present study, presenteeism is operationalized as health‐related work functioning and measured using the Stanford Presenteeism Scale (SPS‐6).

The theoretical model of this study is depicted in Figure 1.

## 2 Method

### 2.1 Procedure and Participants

The present study employed a three‐wave longitudinal design, assessing all focal variables at each measurement point. Data collection occurred at three distinct intervals: September 2024, February 2025, and May 2025, with approximately three months between each wave. This design was implemented to investigate the temporal relationships among the study variables and to enhance causal inferences within the proposed mediation model (Cole and Maxwell 2003).

Participants were recruited via Prolific (Palan and Schitter 2018), an online platform designed for research recruitment that offers access to a diverse range of adult samples. The study aimed to include employees from various occupational sectors, encompassing both blue‐collar and white‐collar professions. Prior to the commencement of the survey, participants received an information statement detailing the study's objectives, procedures, potential risks, confidentiality measures, the voluntary nature of participation, and participants' right to withdraw at any point without penalty. Informed consent was obtained electronically from participants before they accessed the survey questions.

Participants received compensation via the Prolific platform, in accordance with Prolific's payment standards. Compensation was allocated separately for each completed survey wave, with eligibility for subsequent waves contingent on completing the preceding survey. No additional incentives were offered beyond the approved compensation for participation.

Eligibility criteria mandated that participants be at least 18 years of age, employed full‐time (minimum of 30 hours per week), and fluent in English. To be included in the longitudinal analyses, participants were required to complete all three waves of data collection. Exclusion criteria encompassed failure to meet eligibility requirements, incomplete participation in the survey waves, provision of incomplete responses, and failure to pass data‐quality checks. To enhance the validity of responses, attention‐check items were incorporated within the surveys; responses indicating insufficient engagement or inconsistent answering patterns were subsequently excluded from analysis.

Data were collected anonymously through the Prolific platform and securely stored in compliance with the University's research data management protocols. The analytical dataset did not include any personally identifiable information. Participant identifiers obtained from Prolific were utilized exclusively for longitudinal matching across survey waves and were subsequently removed from the final analytical dataset to ensure confidentiality. Given that the study encompassed health‐related variables, including presenteeism, participants were informed that their responses would be analyzed confidentially and reported solely in aggregate form.

Among the original 504 participants at Time 1, 444 continued to participate in Wave 2, and 342 completed all three waves of the study by Time 3, resulting in an overall retention rate of 67.9%. To evaluate potential attrition bias, participants who completed all three waves were compared with those who did not complete all three waves on demographic characteristics and key study variables measured at Time 1. Independent‐samples *t*‐tests were conducted for continuous variables, utilizing Welch's *t*‐test when the assumption of equal variances was not satisfied. For categorical variables, Pearson chi‐square (*χ* ^2^) tests were employed. As indicated in Supporting Information S1: Tables, specifically Supporting Information S1: Table S1, none of the comparisons for continuous variables reached statistical significance (all adjusted ps > 0.05), and no demographic comparisons among categorical variables were statistically significant (all ps > 0.05). These findings provide no statistically significant evidence of systematic attrition bias between participants who completed all three waves and those who did not, thereby supporting the comparability of the longitudinal sample with the original Time 1 sample.

Demographic analysis revealed that 51.5% identified as male, 48.2% as female, and 0.3% preferred not to disclose their gender, with the majority (76.4%) falling within the 20‐ to 50‐year age range. Marital status revealed that 50.6% of respondents were married, 41.5% were single, and 8% were either divorced, separated, or widowed. Employment status indicated that 70% of respondents were engaged in white‐collar occupations, 18.5% in blue‐collar jobs, and 11.5% represented a combination of both sectors. Educationally, 84.1% held university degrees. Job metrics revealed an average tenure of 8.39 years, a working schedule of 38.55 hours per week, and an average of 2.56 hours of overtime per week.

### 2.2 Measures

*Job demands* were assessed using a five‐item scale developed by Boyar et al. (2007), which evaluates individuals' perceptions of job demands. Participants responded on a five‐point Likert scale, ranging from 1 (strongly disagree) to 5 (strongly agree), with higher scores indicating a greater perceived job demand (Boyar et al. 2007). An example item is: ‘My job requires all of my attention’ (*α* = 0.91).

*Family demands* were measured using the PFD scale, also created by Boyar et al. (2007), consisting of four items that capture perceptions of family‐related obligations. Participants rated their agreement on a five‐point Likert scale, ranging from 1 (‘strongly disagree’) to 5 (‘strongly agree’). An illustrative item was: ‘I have to work hard on family‐related activities’ (*α* = 0.91).

*Safety Leadership* comprised assessments of SSTL and SSPL, utilizing 10‐item and 3‐item scales adapted from Barling et al. (2002), and J. E. Mullen and Kelloway (2009). Participants rated their responses using a five‐point Likert scale, with *α* values of 0.95 for SSTL and 0.81 for SSPL.

*Presenteeism* was assessed using the Stanford Presenteeism Scale (SPS‐6) (Koopman et al. 2002). The SPS‐6 measures employees' perceptions of the impact of health issues on their ability to concentrate, complete work tasks, and sustain productivity in the workplace (Koopman et al. 2002). Accordingly, in the present study, presenteeism is conceptualized as health‐related work functioning rather than as sickness attendance, attendance pressure, or withdrawal behavior. This six‐item scale encompasses work completion and distraction avoidance. The SPS‐6 employs a five‐point Likert scale, with response options ranging from 1 (‘strongly disagree’) to 5 (‘strongly agree’). Items 1, 3, and 4 were reverse‐scored before calculating the total SPS‐6 score. The total score ranges from 6 to 30, with higher scores indicating better health‐related work functioning, reflecting a greater ability to concentrate on and complete work despite health problems. Accordingly, throughout the empirical analyses, higher SPS‐6 scores represent better health‐related work functioning, and the term presenteeism refers to this SPS‐6‐operationalized construct (*α* = 0.81).

*Psychosocial Safety Climate (PSC)* was evaluated using the PSC‐12 (Hall et al. 2010), which includes 12 items rated on a five‐point scale, resulting in scores from 12 to 60 (*α* = 0.97).

*Safety behavior* was assessed through two components of safety behavior: *SC* and SP (Neal et al. 2000). Participants rated their behaviors on a Likert scale from 1 (never) to 5 (always). Example items for safety compliance include, ‘I utilize appropriate safety procedures for my job,’ and for safety participation, ‘I exert additional effort to enhance workplace safety’ (*α* = 0.91 for SC, 0.90 for SP).

*Safety motivation* was assessed via a three‐item scale (Neal and Griffin 2006), with responses on a five‐point Likert scale, where one indicated ‘strongly disagree’, and five indicated ‘strongly agree.’ An example statement is: ‘I see value in making an effort to uphold or enhance my personal safety’ (*α* = 0.86).

### 2.3 Statistical Analysis

The data were analyzed using descriptive statistics, correlation assessments, and reliability evaluations with IBM SPSS Statistics 30. Longitudinal model testing employed Structural Equation Modeling (SEM) via IBM SPSS AMOS 30. This methodology facilitated the examination of inter‐ and intra‐variable relationships, enabling the testing of hypotheses regarding the moderating and mediating effects of health‐related work functioning (operationalized using the SPS‐6) and PSC within the JD‐R‐OHS model. Interaction terms representing each hypothesized moderation relationship were computed by multiplying the relevant predictor and moderator variables and were entered into the SEM models to test the proposed conditional effects. Significant interactions were interpreted using simple‐slopes analyses. To evaluate the hypotheses, moderation and mediation analyses were used on 5000 bootstrap samples (Hayes 2013). Here, moderation and mediation refer to instances where the relationship between a predictor independent variable, such as PJD, and a dependent outcome variable, such as SC or SM, varies based on the levels of intervening variables, influenced by mediators or moderators such as health‐related work functioning (SPS‐6) and PSC. To preserve temporal ordering within the hypothesized JD‐R‐OHS model, variables were entered into the structural equation model according to their designated measurement occasions. Although data were collected across three waves, not all constructs were modeled as repeated measures. Specifically, perceived PSC and health‐related work functioning (SPS‐6) were operationalized as temporally specific mediators and entered the analyses using only Time 2 (T2) data. Consequently, autoregressive paths were not estimated for these constructs because repeated measurements were not incorporated into the final SEM specification. This modeling approach maintained temporal separation between predictors, mediators, and outcomes and aligned with the study's conceptual framework.

The adequacy of models was assessed using a chi‐square difference test, incorporating absolute, incremental, and parsimony fit models. Fit indices such as the Goodness of Fit Index (GFI), Comparative Fit Index (CFI), and Tucker‐Lewis Index (TLI) establish a threshold of 0.90 for acceptable fit (Byrne 2013), while values above 0.80 are acceptable for the Relative Fit Index (RFI) and Normed Fit Index (NFI) (Khairi et al. 2021). For the RMSEA, values below 0.05 indicate a close fit, with up to 0.08 considered acceptable (Byrne 2013). Furthermore, a Standardised Root Mean Square Residual (SRMR) below the 0.08 threshold indicates a good fit between the hypothesized model and the observed data (Hu and Bentler 1999). Control variables, including age, gender, job experience, and education, were included to enhance accuracy (Fukuzaki and Iwata 2023; Mazzetti et al. 2023).

Residual covariances were controlled for between each respective safety criteria variable at Time 1 and Time 3 to account for temporal dependencies in repeated measures. Specifically, the residuals of SC, SP, and SM were correlated across time (Little 2024), thereby adjusting for shared variance not explained by the latent constructs or predictors and reducing potential bias in parameter estimates from unmodeled autocorrelations. Model fit indices and path estimates were evaluated with these specified residual correlations.

Because all focal constructs were measured using self‐report questionnaires, procedural and statistical steps were undertaken to minimize the risk of common method variance (CMV). Procedurally, the study employed a three‐wave longitudinal design that temporally separated the measurement of predictors, mediators, and outcomes. Participants were assured of anonymity and confidentiality, reducing evaluation apprehension and socially desirable responding. Established and psychometrically validated instruments were used throughout the study.

Statistically, the temporal separation of measurements substantially reduces the likelihood that observed relationships are solely attributable to common method bias. Nevertheless, because all variables were ultimately obtained from the same respondents, CMV cannot be entirely excluded. Accordingly, the findings should be interpreted with appropriate caution, particularly those involving relatively small or unexpected effects.

## 3 Results

Table 1 summarizes the means, standard deviations, and intercorrelations of the variables analyzed in this study. Results indicated a positive correlation between PJD and PFD, while SSTL showed a negative association with SSPL. Health‐related work functioning (SPS‐6) exhibited small but statistically significant relationships with PFD, PSC, and safety outcomes. Additionally, PSC demonstrated strong positive correlations with SSTL and negative correlations with SSPL, alongside noteworthy positive relationships with safety compliance and motivation across different time points. The safety outcomes assessed at Time 1 and Time three exhibited moderate to strong interrelations, suggesting stability across measurement occasions. All correlations aligned with theoretical expectations, with several attaining statistical significance.

**TABLE 1:** Means, standard deviations, and correlations of the study variables.

Variables	*M*	SD	1	2	3	4	5	6	7	8	9	10	11
1. PJD	20.3	3.7											
2. PFD	12.6	4.1	0.31**										
3. SSTL	34.3	8.8	0.03	0.15**									
4. SSPL	7.1	2.9	0.02	0.04	−0.60**								
5. HWF	22.0	4.9	−0.07	−0.11*	0.04	−0.11*							
6. PSC	38.8	11.3	−0.03	−0.03	0.57**	−0.42**	0.21**						
7. SC time 3	12.3	2.1	0.07	0.09	0.21**	−0.17**	0.15**	0.24**					
8. SP time 3	10.1	2.9	0.12*	0.19**	0.35**	−0.21**	0.09	0.37**	0.48**				
9. SM time 3	12.9	1.6	−0.03	0.01	0.11*	−0.15**	0.20**	0.18**	0.57**	0.31**			
10. SC time 1	12.3	1.9	0.08	0.10	0.29**	−0.27**	0.13*	0.27**	0.51**	0.35**	0.39**		
11. SP time 1	10.2	2.8	0.09	0.20**	0.43**	−0.28**	0.07	0.33**	0.32**	0.65**	0.22**	0.50**	
12. SM time 1	13.0	1.6	0.07	0.08	0.20**	−0.19**	0.22**	0.23**	0.45**	0.25**	0.62**	0.41**	0.27**

*Note: N* = 342.

Abbreviations: HWF, Health‐related work functioning (SPS‐6), Higher scores indicate better health‐related work functioning; M, means; PFD, Perceived Family Demands; PJD, Perceived Job Demands; PSC, Psychosocial Safety Climate; SC, Safety Compliance; SD, standard deviations; SM, Safety Motivation; SP, Safety Participation; SSPL, Safety‐Specific Passive Leadership; SSTL, Safety‐Specific Transformational Leadership.

^*^ *p* < 0.05.

^**^ *p* < 0.01.

The structural model demonstrated acceptable overall fit to the data (*χ* ^2^/df = 1.62, RMSEA = 0.043, SRMR = 0.07, GFI = 0.93, and AGFI = 0.91). For incremental fit parameters, CFI = 0.94, IFI = 0.94, TLI = 0.93, RFI = 0.84, and NFI = 0.85 surpassed commonly recommended thresholds (Byrne 2013; Khairi et al. 2021). The parsimonious fit parameters, PCFI = 0.87, PGFI = 0.83, PNFI = 0.78, each exceeded the 0.50 minimum threshold (Browne and Cudeck 1992). These findings suggest a satisfactory fit of the data with the research model. The proposed model effectively represented the observed covariance structure. However, an acceptable global model fit should not be construed as conclusive evidence supporting all hypothesized structural relationships. The evaluation of support for the proposed hypotheses was based on the significance and directionality of the individual structural pathways and the indirect effects.

To enhance the clarity of the complex hypothesis framework, a summary of the evidence supporting all proposed hypotheses is presented in the Supporting Information S1: Table S2. A structural equation analysis investigated whether the hypothesized effects of JD and JR on safety outcomes are mediated and moderated by presenteeism and PSC. Tables 2 and 3 present the direct effects of JD and JR on the mediators and their impact on safety outcomes. In Table 2, PJD lacked a significant association with health‐related work functioning (SPS‐6) (H1a1; *β* = −0.161, 95% CI [−0.450, 0.088], *p* = 0.262) or PSC (H1a2; *β* = −0.045, 95% CI [−0.229, 0.148], *p* = 0.647). Similarly, PFD did not significantly predict health‐related work functioning (SPS‐6) (H1a1; *β* = −0.124, 95% CI [−0.254, 0.006], *p* = 0.102) or PSC (H1a2; *β* = −0.092, 95% CI [−0.187, 0.001], *p* = 0.077).

**TABLE 2:** Direct effects of predictors on the mediators and mediators within the job demands‐resources‐occupational health and safety (JD‐R‐OHS) model.

Predictors	HWF				Psychosocial safety climate			
	*β*	95% CI		*p*	*β*	95% CI		*p*
		LL	UL			LL	UL	
PJD	−0.161	−0.450	0.088	0.262	−0.045	−0.229	0.148	0.647
PFD	−0.124	−0.254	0.006	0.102	−0.092	−0.187	0.001	0.077
SSTL	0.048	−0.152	0.264	0.693	0.493	0.333	0.652	< 0.001
SSPL	−0.514	−0.893	−0.223	0.003	−0.537	−0.939	−0.218	0.004

*Note:* HWF, Health‐related work functioning (SPS‐6); PJD, Perceived Job Demands; PFD, Perceived Family Demands; SSTL, Safety‐Specific Transformational Leadership; SSPL, Safety‐Specific Passive Leadership.

**TABLE 3:** Direct effects of predictors on the safety outcomes in the job demands‐resources‐occupational health and safety (JD‐R‐OHS) model.

Predictors	Safety compliance				Safety participation				Safety motivation			
	*β*	95% CI		*p*	*β*	95% CI		*p*	*β*	95% CI		*p*
		LL	UL			LL	UL			LL	UL	
PJD	0.048	−0.113	0.206	0.543	0.054	−0.111	0.215	0.549	−0.024	−0.143	0.077	0.708
PFD	0.024	−0.054	0.098	0.620	0.075	−0.015	0.160	0.121	−0.015	−0.077	0.042	0.655
SSTL	0.036	−0.095	0.169	0.614	0.053	−0.097	0.210	0.514	0.077	−0.073	0.131	0.575
SSPL	0.155	−0.122	0.472	0.267	0.053	.‐0.174	0.065	0.321	−0.086	−0.416	0.166	0.429
HWF	0.015	−0.046	0.080	0.674	−0.020	−0.090	0.049	0.611	−0.006	−0.058	0.045	0.838
PSC	0.093	0.011	0.145	0.050	0.232	0.116	0.355	< 0.001	0.075	0.012	0.142	0.048

Abbreviations: HWF, Health‐related work functioning (SPS‐6); PFD, Perceived Family Demands; PJD, Perceived Job Demands; PSC, Psychosocial Safety Climate; SC, Safety Compliance; SM, Safety Motivation; SP, Safety Participation; SSPL, Safety‐Specific Passive Leadership; SSTL, Safety‐Specific Transformational Leadership.

With respect to Hypothesis 1b, while SSTL did not significantly correlate with health‐related work functioning (SPS‐6) (H1b1; *β* = 0.048, 95% CI [−0.152, 0.264], *p* = 0.693), it exhibited a significant positive relationship with PSC (H1b2; *β* = 0.493, 95% CI [0.333, 0.652], *p* < 0.001). In contrast, SSPL showed significant negative relationships with both health‐related work functioning, (H1b3; *β* = −0.514, 95% CI [−0.893, −0.223], *p* = 0.003) and PSC (H1b4; *β* = −0.537, 95% CI [−0.939, −0.218], *p* = 0.004). This indicates that higher SSPL was associated with poorer health‐related work functioning and lower PSC.

Table 3 results indicated that Hypotheses H2b (1 and 2) were unsupported, as neither SSTL nor SSPL showed statistically significant direct associations with SC, SP, or SM. Specifically, SSTL showed non‐significant associations with SC (*β* = 0.036, *p* = 0.614), SP (*β* = 0.053, *p* = 0.514), and SM (*β* = 0.077, *p* = 0.575). SSPL likewise showed non‐significant associations with SC (*β* = 0.155, *p* = 0.267), SP (*β* = 0.053, *p* = 0.321), and SM (*β* = −0.086, *p* = 0.429). Furthermore, Hypothesis H3a was not supported; higher health‐related work functioning was not significantly positively associated with any of the safety outcomes: safety compliance (*β* = 0.015, *p* = 0.674), safety participation (*β* = −0.020, *p* = 0.611), or safety motivation (*β* = −0.006, *p* = 0.838). Conversely, PSC was positively associated with SP (*β* = 0.232, 95% CI [0.116, 0.355], *p* < 0.001) and SM (*β* = 0.075, 95% CI [0.012, 0.142], *p* = 0.048). The association between PSC and SC was marginally significant, being at the conventional threshold for statistical significance (*β* = 0.093, 95% CI [0.011, 0.145], *p* = 0.050). These findings are consistent with prior research reporting positive associations between PSC and safety outcomes, and provide support for Hypothesis 3b with respect to safety participation and safety motivation. The association with safety compliance sat at the conventional significance threshold and is therefore interpreted with caution.

Table 4 examines indirect effects, specifically the sequential mediating role of PSC in Hypothesis 4b2 and health‐related work functioning in Hypothesis 4a2. Additionally, Supporting Information S1: Table S3 presents comprehensive results for all indirect effects. As predicted, SSTL demonstrated significant positive indirect effects on SP (*β* = 0.08, *p* < 0.001), SM (*β* = 0.025, *p* = 0.030), and SC (*β* = 0.034, *p* = 0.045) through PSC. Conversely, SSPL showed significant negative indirect effects on SP (*β* = −0.110, *p* = 0.001), SM (*β* = −0.03, *p* = 0.029), and SC (*β* = −0.041, *p* = 0.033) via PSC, providing empirical support for Hypothesis 4b2. These findings provide the strongest support for the proposed PSC‐mediated pathway, although the direct association between PSC and safety compliance was comparatively weaker. Hypotheses 4a1 and 4b1, proposing indirect effects of PJD and PFD on safety outcomes, were unsupported and excluded from the table. Health‐related work functioning, operationalized using the SPS‐6, did not significantly mediate the relationships between perceived job demands, family demands, or safety‐specific leadership and the safety outcomes. These results indicate that health‐related work functioning was not supported as a robust explanatory mechanism in the present model.

**TABLE 4:** Hypothesis testing of indirect effects, the mediating roles of presenteeism and PSC in the JD‐R‐OHS model.

Effect	*p*	*β*	95% CI		Direction
			LL	UL	
SSTL → PSC → SP	< 0.001	0.08	0.040	0.140	Positive
SSTL → PSC → SM	0.030	0.025	0.006	0.054	Positive
SSTL → PSC → SC	0.045	0.034	0.010	0.072	Positive
SSPL → PSC → SP	0.001	−0.110	−0.228	−0.049	Negative
SSPL → PSC → SM	0.029	−0.03	−0.086	−0.007	Negative
SSPL → PSC → SC	0.033	−0.041	−0.114	−0.010	Negative

*Note:* All hypothesized mediation effects, including non‐significant pathways, are reported with their estimates, *p*‐values, and 95% confidence intervals in Supporting Information S1: Table S2.

Abbreviations: PSC, Psychosocial Safety Climate; SC, Safety Compliance; SM, Safety Motivation; SP, Safety Participation; SSPL, Safety‐Specific Passive Leadership; SSTL, Safety‐Specific Transformational Leadership.

Table 5 presents the results of hypothesis testing within the JD‐R‐OHS framework concerning Hypotheses 5 and 6. Additionally, Supporting Information S1: Table S4 presents the results of hypothesis testing for all indirect effects associated with the study hypotheses within the JD‐R‐OHS framework. A significant interaction was found between job demands and health‐related work functioning (SPS‐6) in predicting safety compliance (*β* = −0.122, *p* = 0.012). Simple‐slope analyses indicated that job demands were more negatively associated with safety compliance among employees reporting poorer health‐related work functioning than among those reporting better functioning. Thus, higher health‐related work functioning appeared to buffer the negative association between job demands and safety compliance, supporting H5a. The interaction between health‐related work functioning and safety leadership was not significant; therefore, H5b was not supported.

**TABLE 5:** Hypothesis testing of direct and moderation effects in the moderating model of the JD‐R‐OHS framework.

Predictor/Interaction				95% CI	
Direct effects	DV	*β*	*p*	LL	UL
Perceived family demands	SP	0.160	0.003	0.066	0.253
Safety‐specific transformational leadership	SP	0.168	0.018	0.024	0.307
Psychosocial safety climate	SC	0.193	0.004	0.074	0.312
	SP	0.301	< 0.001	0.172	0.441
	SM	0.148	0.029	0.025	0.257
Health‐related work functioning	SM	0.170	0.002	0.073	0.269
Moderation Effects (Interactions)					
Perceived job demands × health‐related work functioning	SC	−0.122	0.012	−0.214	−0.016

*Note:* All hypothesized direct and moderation effects, including non‐significant pathways, are reported with their estimates, *p*‐values, and 95% confidence intervals in Supporting Information S1: Table S3.

Abbreviations: DV, Dependent Variable; SC, Safety Compliance; SM, Safety Motivation; SP, Safety Participation.

Regarding direct effects, PSC emerged as a significant positive predictor of all three safety outcomes: SC (*β* = 0.193, *p* = 0.004), SP (*β* = 0.301, *p* < 0.001), and SM (*β* = 0.148, *p* = 0.029). These findings underscore the essential role of PSC in promoting occupational safety, supporting Hypothesis 4b2. SSTL was significantly associated only with SP (*β* = 0.168, *p* = 0.018). Additionally, in the moderated model including the interaction term(s), higher health‐related work functioning was positively associated with SM (*β* = 0.170, *p* = 0.002). This association indicates that employees reporting better functioning while managing health problems also reported greater safety motivation. Because the coefficient was estimated in a model containing the interaction term, it represents the association at the reference level of the moderator and should not be interpreted as a general causal effect. Employees who report higher levels of health‐related work functioning despite health problems may demonstrate greater awareness of safety risks and stronger motivation to engage in safe work practices.

PFD positively correlated with SP (*β* = 0.160, *p* = 0.003), suggesting that employees with greater family demands engage more in discretionary safety behaviors. Hypotheses 6a and 6b proposing moderating effects of PSC were not supported, as no significant interactions involving PSC were observed. Among the control variables, gender emerged as a significant predictor of health‐related work functioning, with male employees reporting higher SPS‐6 scores than female employees (*β* = 0.382, *p* = 0.001). Given that higher SPS‐6 scores indicate better health‐related work functioning, this finding suggests that male employees reported better work functioning while managing health problems. This difference should not be interpreted as evidence of gender differences in the tendency to attend work while unwell, as the SPS‐6 does not assess sickness attendance or attendance decisions.

## 4 Discussion

The aim of this study was to assess the effects of PJD, PFD, SSTL, and SSPL on health‐related work functioning, as a representative of presenteeism, and PSC over time among a heterogeneous sample of workers. This study also evaluated the mediating and moderating roles of health‐related work functioning and PSC in relation to key safety outcomes for these employees.

While several pathways achieved statistical significance, the effect sizes for many were modest. Consequently, it is essential to interpret these findings in terms of both statistical and practical significance. Notably, the indirect effects on presenteeism were predominantly small, suggesting that health‐related work functioning serves as a limited explanatory mechanism within the proposed JD‐R‐OHS framework. In contrast, PSC demonstrated more consistent and significantly stronger associations with leadership and safety outcomes, indicating that organisational climate functions as a more impactful mechanism for enhancing occupational health and safety.

The study indicates that both PJD and PFD were unrelated to PSC or health‐related work functioning, with no indirect effects identified through these mediators. Notably, PFD positively correlated with SP, suggesting that certain family‐related pressures may foster a sense of responsibility that encourages proactive engagement in workplace safety. This trend likely reflects the tendency of individuals with significant family obligations to prioritize behaviors that safeguard their well‐being. When familial considerations intensify, support for family members motivates employees to persist in high‐risk environments, such as underground mining (Yu et al. 2020), positions with limited intrinsic rewards (Menges et al. 2017), and adverse conditions typical for migrant workers (F. Yang et al. 2022). Furthermore, familial concerns serve as a rationale for protecting employees from anxiety and OHS risks in the workplace, reflecting introjected regulated motivation to engage in safety behaviors for external benefits or to alleviate anxiety associated with family responsibilities (Zhou et al. 2025).

Within the health impairment pathway of the JD‐R‐OHS model, health‐related work functioning did not mediate the effects of PJD, PFD, or job resources on safety outcomes. Within the health‐impairment pathway, health‐related work functioning did not mediate the relationships between PJD or PFD and safety outcomes, nor did it mediate the relationships between safety leadership and safety outcomes. Gender was associated with health‐related work functioning, with male employees reporting higher SPS‐6 scores than female employees. Because higher SPS‐6 scores indicate better health‐related work functioning, this result reflects a gender difference in reported functioning while managing health problems. It does not indicate differences in sickness attendance decisions or attendance pressure.

Health‐related work functioning moderated the relationship between job demands and safety compliance. Simple‐slope analyses indicated that job demands were more negatively associated with safety compliance among employees reporting poorer health‐related work functioning than among those reporting better functioning. Thus, better health‐related work functioning appeared to buffer the adverse association between job demands and safety compliance. This finding concerns employees' functioning while managing health problems and should not be interpreted as evidence that sickness attendance affects safety compliance. To our knowledge, the conditional role of health‐related work functioning in safety behavior and motivation has received little empirical attention, and no prior study has tested this moderating effect of job demands on safety outcomes. Evidence from related domains suggests that health‐related functioning associated with presenteeism may serve as a significant boundary condition. Previous research has examined the relationship between health‐related functioning, presenteeism, and performance (Junça‐Silva et al. 2022), but these findings are not directly comparable because studies differ in whether higher scores represent better functioning or greater productivity loss. The findings suggest that employees reporting poorer health‐related work functioning may be less able to maintain safety compliance when job demands are high. This reduced capacity contributes to less effective safety management practices, including inadequate training, limited worker involvement, ineffective communication, insufficient management commitment, suboptimal promotion policies, and noncompliance with regulations. Each factor is a well‐established antecedent to increased risk of occupational accidents (Ajmal et al. 2022; Neal and Griffin 2006; X. Yang et al. 2021).

Despite its moderating effect on safety compliance, health‐related work functioning was positively correlated with safety motivation. According to the conventional SPS‐6 scoring, this finding suggests that employees who report better health‐related work functioning while managing health challenges also exhibit higher levels of safety motivation. This correlation aligns with the perspective that employees who are more capable of functioning effectively at work while addressing health issues are likely to exhibit increased motivation to engage in safe work practices. However, it is important to note that both health‐related work functioning and safety motivation were evaluated through self‐report questionnaires, which may introduce common method variance as a contributing factor to the observed association.

Managers play a critical role in balancing job demands and resources (Schaufeli 2015). Leadership is conceptualized as a higher‐order job resource distinct from individual‐level resources (Tummers and Bakker 2021). Moreover, in the context of safety outcomes, leadership is a key predictor, ranking among the most important job resources (Nahrgang et al. 2011). Within the leadership‐PSC‐OHS pathway, leaders' safety behaviors shape workers' perceptions of the safety climate; transformational leadership is positively associated with PSC, whereas passive leadership is negatively associated. Leaders who prioritize psychosocial health and implement supportive policies foster a safety‐oriented climate, reducing unsafe behaviors and positively influencing job satisfaction, work engagement, and the psychosocial work environment, ultimately contributing to lower accident rates (Boamah et al. 2018; Ree and Wiig 2020). Additionally, leaders’ safety behavior may enhance worker safety performance, including SP and adherence to safety procedures (Griffin and Hu 2013).

When managers adopt a passive leadership style characterized by limited action and responsiveness towards workplace safety, their approach becomes ineffective as it is reactive rather than proactive. Such leadership provides little direction or purpose regarding safety (J. E. Mullen and Kelloway 2009). Organisations led by passive managers often experience elevated safety‐related events due to a diminished safety climate resulting from leaders' failure to promote safe behaviors. Consequently, neglect of safety initiatives weakens PSC, increases occupational health risks, and deteriorates organisational performance (Smith et al. 2016). Interestingly, within the Leadership–Presenteeism–OHS pathway, a negative relationship was observed between SSPL and SPS‐6 health‐related work‐functioning scores. Given that health‐related work functioning was measured using the SPS‐6, this finding should be interpreted as an association between passive leadership and employees’ health‐related work functioning while managing health problems, rather than as evidence that passive leadership influences employees' decisions to attend work despite illness. The negative association between SSPL and health‐related work functioning is consistent with the theoretical proposition that passive leadership may undermine employees' ability to function effectively at work when experiencing health problems. Thus, rather than indicating an effect on sickness attendance, the finding suggests that employees exposed to passive safety leadership may experience poorer health‐related work functioning when working while managing health problems. Nevertheless, because the SPS‐6 does not directly assess sickness attendance or attendance decisions, this finding should not be interpreted as evidence regarding whether employees attend work while unwell.

The findings indicate that PSC represented the most consistently supported organisational pathway within the proposed JD‐R‐OHS model. While the overall model received mixed empirical support, PSC provided the clearest evidence as a mechanism linking safety‐specific leadership with safety outcomes, particularly safety participation and safety motivation. The PSC‐mediated pathways from both safety‐specific transformational and passive leadership to safety participation, safety motivation, and safety compliance were statistically significant, whereas the direct association between PSC and safety compliance was comparatively weaker. In contrast, the hypothesized pathways involving perceived job demands, perceived family demands, and health‐related work functioning received comparatively limited validation. These results align with previous research that demonstrates a positive association between a supportive PSC and enhanced employee compliance with safety procedures and increased safety participation (Dera et al. 2025; Mirza et al. 2022). Thus, PSC appears to function as the principal mediating mechanism through which leadership and psychosocial organisational resources relate to safety outcomes in the present study. When organisational efforts foster high PSC levels, employees may be more likely to commit to safe task performance and adhere to safety policies. Additionally, managerial efforts to cultivate a psychosocially safe climate are correlated with greater compliance with safety protocols, reduced accidents, and the promotion of safe behaviors at work. Employees' perception of the organisation's proactive guidance in promoting safety behaviors may increase their compliance with regulations and participation in safety‐related activities (Pilbeam et al. 2016). Research indicates that perceived safety climate mediates the relationship between safety leadership and compliance (Clarke 2006; Omidi et al. 2023). These findings underline the impact of PSC‐oriented programs on enhancing safety behavior and supporting overall OHS outcomes.

The current findings are consistent with contemporary perspectives that conceptualize PSC as a strategic organisational resource that enhances psychosocial risk management, rather than merely an employee perception of workplace climate. Within this framework, PSC embodies organisational systems that prioritize psychological health and safety through management commitment, effective communication, and employee involvement. This expanded interpretation aligns with recent advancements, which advocate integrating psychosocial risk management into organisational safety systems (Mirza et al. 2022; Yu et al. 2022; W. Liu et al. 2025).

### 4.1 Theoretical Implications

This study provides evidence that is consistent with extending JD–R theory in the OHS domain by distinguishing the roles of climate‐level and individual‐level mechanisms. Whereas PSC appeared to be a robust explanatory pathway, the largely unsupported hypotheses involving health‐related work functioning require a more refined interpretation of the health impairment process within the JD–R framework. Contrary to core JD–R assumptions and the proposed mediation hypotheses, health‐related work functioning did not function as a general mediating mechanism linking job demands or leadership resources to safety outcomes. These null findings suggest that presenteeism may not represent a universal indicator of resource depletion or strain in safety‐relevant contexts. Instead, the results indicate important boundary conditions regarding when health‐related difficulties translate into impaired work functioning and safety‐relevant outcomes. The present findings suggest that the role of presenteeism within the JD–R–OHS framework may be more context‐dependent than originally hypothesized. Rather than functioning as a central explanatory mechanism, health‐related work functioning may operate as a conditional factor whose effects vary across organisational contexts and measurement approaches.

Moreover, presenteeism moderated the relationship between job demands and safety compliance but did not consistently mediate effects across the health impairment pathway. This pattern suggests that health‐related work functioning did not function as a general mediating mechanism but did moderate the relationship between job demands and safety compliance. This pattern suggests that health‐related work functioning may operate as a conditional factor whose protective role becomes more apparent when employees face high job demands. The finding also reinforces the importance of distinguishing the SPS‐6 measure of functioning while managing health problems from measures of sickness attendance.

In contrast, PSC emerged as the most consistently supported organisational resource, particularly through its mediating role in the relationships between safety‐specific leadership and safety participation and motivation. Although the PSC‐mediated pathway to safety compliance was also statistically significant, the direct association between PSC and safety compliance was comparatively weaker. This pattern suggests that climate‐level resources may represent important organisational mechanisms for safety outcomes, while also indicating that their effects may vary across specific safety outcomes. These findings suggest that climate‐level resources may represent more stable and proximal mechanisms for safety outcomes than individual health behaviors, underscoring the need for clearer theoretical differentiation within JD–R–OHS models.

Importantly, the present findings indicate that the theoretical contribution of the proposed JD‐R‐OHS framework lies not solely in providing uniform support for all hypothesized relationships, but in identifying PSC as the most consistently supported organisational mechanism linking safety‐specific leadership to safety outcomes. While the JD‐R framework provides an appropriate theoretical foundation, the results suggest that organisational climate resources received stronger empirical support than the proposed presenteeism pathway in explaining safety motivation and safety behaviors. Consequently, these findings refine, rather than fully validate, the proposed model by emphasizing which mechanisms receive the strongest empirical support.

### 4.2 Practical Implications

The practical implications primarily arise from the consistent findings regarding PSC and safety‐specific leadership. Although several statistically significant pathways were identified, the effect sizes suggest that organisational interventions should prioritize mechanisms that demonstrate the greatest practical impact. In this study, PSC demonstrated the most consistently supported relationships with safety outcomes, particularly safety participation and safety motivation, and provided significant mediating pathways between safety‐specific leadership and all three safety outcomes. Accordingly, organisational efforts to strengthen the psychosocial safety climate through safety‐specific leadership, effective communication, and psychosocial risk management may support improvements in workplace safety. Training that emphasizes open communication, management commitment to employee well‐being, and participatory decision‐making may help cultivate a stronger PSC, which may improve employees' perceptions of safety support and prompting proactive safety behaviors. Investing in such training not only may reinforce the organisation's values concerning psychological health and safety but also may help ensure that supervisory practices align with broader organisational safety priorities. The present findings provide the strongest support for PSC as an organisational resource, particularly in relation to safety participation and safety motivation, while the evidence concerning safety compliance was comparatively weaker. Furthermore, PSC represented the most consistently supported mediating mechanism through which safety‐specific leadership was associated with safety outcomes. Therefore, interventions designed to enhance PSC through effective leadership practices are likely to yield the most substantial organisational benefits.

Furthermore, the current findings provide limited support for interventions to alleviate the strain associated with job demands, particularly in workplaces where employees experience health‐related difficulties that may affect their work functioning. While perceived job demands were not directly linked to PSC or presenteeism, better health‐related work functioning buffered the negative association between job demands and safety compliance. Consequently, organisations may benefit from implementing strategies such as workload management, flexible scheduling, and supportive absence policies in environments where high job demands coexist with poorer health‐related work functioning. These approaches may help mitigate safety risks associated with reduced health‐related work functioning under conditions of high job demands. However, these implications should be interpreted with caution because the study measured perceived functioning rather than objective productivity loss or sickness absence.

Balancing SL strategies is essential to engage employees in safety participation without reinforcing unhealthy attendance norms. Leaders must model and support safety compliance alongside self‐care behaviors, helping create a psychologically safe environment where employees prioritize their health without fear of reprisal or stigma. The findings of the present study indicate that supportive leadership and a PSC may foster healthier workplace norms. However, this study does not provide direct evidence that leadership interventions improve health‐related work functioning. Consequently, leadership initiatives should primarily be regarded as strategies to enhance PSC and improve safety outcomes, rather than as direct methods to improve health‐related work functioning.

Integrating these approaches into a robust PSC framework may enhance occupational safety programs by addressing both organisational and individual factors that contribute to safety performance. The practical implications underscore the necessity for organisations to prioritize interventions substantiated by robust empirical evidence, specifically those aimed at enhancing PSC and fostering safety‐specific leadership practices. Recommendations associated with workload management and health‐related work functioning should be regarded as complementary and provisional until further longitudinal and intervention research substantiates their direct effects on occupational health and safety outcomes.

The findings of this study provide partial empirical support for the proposed JD‐R‐OHS framework. Although the structural model demonstrated acceptable overall fit, support for the hypothesized relationships varied. However, rather than offering uniform support for all hypothesized mechanisms, the study consistently identified PSC as the primary organisational pathway linking safety‐specific leadership to safety motivation and safety behaviors. Consequently, these findings refine the proposed framework by underscoring the relative significance of organisational climate resources in comparison to the more limited role of the presenteeism pathway.

### 4.3 Limitations and Future Research Directions

This study has limitations that should be considered when interpreting the findings and guiding future research. While the three‐wave longitudinal design improves causal inference over cross‐sectional studies, it may not fully capture the dynamic temporal processes involved in presenteeism, PSC, and safety outcomes. Future studies could utilize more granular longitudinal designs, such as diary or experience sampling methods, to examine short‐term fluctuations and reciprocal effects. The reliance on self‐reported data introduces the potential for common‐method bias; future research should incorporate objective safety records or multisource ratings to validate findings and enhance the robustness of the results. Although the longitudinal design reduced temporal proximity among variables, all constructs were measured using self‐report instruments, increasing the possibility that common method variance and socially desirable responding influenced some observed relationships. This limitation is particularly relevant for the unexpected associations involving presenteeism. Future studies should incorporate objective safety indicators, supervisor‐rated safety behaviors, sickness absence records, and direct measures of attendance decisions to distinguish health‐related work functioning from attendance behavior and to clarify the mechanisms linking presenteeism with occupational safety outcomes. This research also focused on specific leadership styles and PSC as mediators and moderators, allowing for the exploration of additional job and personal resources within the JD‐R framework. Future investigations should examine how health‐related work functioning, as measured by the SPS‐6, interacts with demands and resources over time, while accounting for individual differences and organisational contingencies. This would enhance understanding of the mechanisms governing occupational safety and employee well‐being.

The present findings should be interpreted in light of several sample characteristics that constrain both generalizability and the validity of inferences drawn from the JD–R–OHS model. The sample was predominantly white‐collar employees (approximately 70%), highly educated, and largely composed of native English speakers across five English‐speaking countries. Additionally, a substantial proportion of participants were single workers (approximately 42%). This demographic profile has important implications for the study's conclusions, particularly regarding work–family demands and occupational safety outcomes. White‐collar roles typically involve lower exposure to acute physical hazards, greater autonomy, and more flexible work arrangements than blue‐collar or high‐risk occupations. As such, the observed relationship among job demands, PSC, presenteeism, and safety behaviors may not fully reflect the dynamics present in high‐hazard environments such as construction, mining, or manufacturing, where physical risks, regulatory pressures, and task structures differ substantially.

Similarly, the high proportion of single participants may have reduced variability in family‐related demands, potentially attenuating the strength of associations involving family demands and PSC. Employees with caregiving responsibilities often experience more complex and chronic work–family pressures, which may interact differently with organisational climate and safety‐related behaviors. Consequently, the restricted demographic variability represents not only a limitation for external validity but also a potential constraint on internal validity, as the reduced heterogeneity may limit the extent to which the mechanisms identified can be attributed to broader JD–R processes rather than characteristics of this specific sample. Future research should replicate and extend the JD–R–OHS model using samples with greater occupational and family‐structure diversity, particularly in blue‐collar and high‐hazard sectors. A longitudinal design across varied hazard profiles would help determine whether the observed effects reflect sample‐specific characteristics or more generalizable contextual contingencies within the JD–R framework.

An additional limitation of the present study pertains to the operationalization of presenteeism through the SPS‐6. This investigation utilized the SPS‐6, which evaluates health‐related work functioning while employees are physically present at work, rather than addressing the behavioral decision to attend work despite being unwell. Because higher SPS‐6 scores indicate better health‐related work functioning, the findings reflect variation in employees' functioning while managing health problems rather than greater or lower sickness attendance. Therefore, the results should be interpreted as reflecting variations in work functioning associated with health issues, rather than as indicating sickness absence behavior. Future research should combine the SPS‐6 with direct measures of sickness attendance, absence, objective productivity, and supervisor‐rated safety behavior to distinguish health‐related work functioning from attendance behavior and productivity loss.

## 5 Conclusion

This study contributes to the JD‐R and occupational health and safety literature by demonstrating that PSC represents the most robust organisational mechanism within the proposed JD‐R‐OHS framework. Although the broader model received mixed empirical support, the findings consistently indicate that PSC emerged as the most consistently supported organisational mechanism in the model, with particularly clear evidence for its role in linking safety‐specific leadership with safety participation and safety motivation. The significant PSC‐mediated pathways to safety compliance also provide support for the motivational pathway, although the direct PSC–safety compliance association was comparatively weaker. In contrast, the hypothesized pathways involving perceived job demands, perceived family demands, and health‐related work functioning received comparatively limited support. Rather than providing uniform support for all proposed mechanisms, the findings refine the JD‐R‐OHS framework by identifying PSC as the principal organisational pathway through which safety‐specific leadership is associated with safety outcomes. The findings suggest that OHS interventions may benefit from shifting system‐based controls to integrated strategies that cultivate PSC, monitor health‐related work functioning, and address work‐family demands as modifiable stressors affecting safety outcomes. Collectively, these findings suggest that PSC represents the more robust organisational mechanism within the proposed JD–R–OHS model, whereas the role of health‐related work functioning requires further theoretical refinement and replication using multi‐method designs before firm conclusions can be drawn.

## Ethics Statement

We confirm that this manuscript is original, has not been published previously, and is not under consideration by any other journal. All authors have approved the manuscript and agree with its submission to Stress and Health. The study received ethical approval from Griffith University (GU Ref No: 2024/132), and all procedures were conducted in accordance with relevant ethical standards.

## Conflicts of Interest

The authors declare that there are no financial or commercial conflicts of interest relating to this work. Professor Paula Brough, who is listed as a co‐author, serves as Deputy Editor of Stress and Health; however, she had no involvement in the editorial decision‐making, peer review, reviewer selection, or handling of this manuscript, and all editorial decisions were made independently by other members of the journal's editorial team.

## Supporting information


Supporting Information S1

## Data Availability

The data that support the findings of this study are available on request from the corresponding author. The data are not publicly available due to privacy or ethical restrictions.
