# Supplementary material for: Uncovering Pathways to Occupational Health and Safety (OHS): Presenteeism and Psychosocial Safety Climate in the JD–R–OHS Model
Source: Stress Health. 2026 Sep 15;42(5):e70222. doi: 10.1002/smi.70222 (PMC13575849; doi:10.1002/smi.70222)
Supplement: Supplementary file 1 [file SMI-42-e70222-s001.docx]

**Supplementary Tables**

**Table S1.**

Attrition Analysis Comparing Three-Wave Completers and Non-Completers at Time 1

| Variable | Three-wave completers (n = 342) M (SD) | Non-completers (n = 162) M (SD) | *t* | df | *p* |
| --- | --- | --- | --- | --- | --- |
| *Demographic characteristics* | | | | | |
| Age | 38.55 (10.87) | 40.51 (11.07) | 1.88 | 316.67 | .558 |
| Job experience | 7.17 (7.06) | 8.56 (8.10) | 1.89 | 286.03 | .558 |
| *Key study variables* | | | | | |
| PJD | 20.24 (3.60) | 19.88 (4.34) | −0.91 | 274.43 | 1.000 |
| PFD | 12.90 (4.06) | 12.30 (4.25) | −1.53 | 502.00 | .756 |
| SSTL | 34.23 (8.40) | 35.37 (9.08) | 1.39 | 502.00 | .820 |
| SSPL | 7.46 (2.83) | 6.80 (2.86) | −2.40 | 319.65 | .170 |
| PSC | 38.27 (11.33) | 40.57 (12.09) | 2.04 | 304.12 | .344 |
| HWF | 21.40 (4.28) | 22.60 (4.99) | 2.64 | 282.14 | .099 |
| Safety motivation | 12.80 (1.60) | 12.89 (1.82) | 0.54 | 287.55 | 1.000 |
| Safety compliance | 12.23 (1.91) | 12.65 (1.99) | 2.28 | 309.85 | .207 |
| Safety participation | 10.12 (2.70) | 10.54 (2.87) | 1.56 | 304.71 | .756 |

**Categorical demographic characteristics**

| **Variable** | **Pearson χ²** | **df** | ***p*** |
| --- | --- | --- | --- |
| Gender | 2.91 | 3 | .405 |
| Education | 6.90 | 6 | .331 |
| Overtime | 20.13 | 20 | .450 |
| Marital status | 2.22 | 20 | .136 |
| Occupation type | 0.06 | 2 | .969 |

*Note.* *M*, mean; *SD*, standard deviation; *t*, independent-samples *t*-test; df, degrees of freedom; χ², Pearson chi-square statistic. *SC*, Safety Compliance; *SP*, Safety Participation; *SM*, Safety Motivation; *PJD*, Perceived Job Demands; *PFD*, Perceived Family Demands; *SSTL*, Safety-Specific Transformational Leadership; *SSPL*, Safety-Specific Passive Leadership; *PSC*, Psychosocial Safety Climate; *HWF*, Health-related Work Functioning.

**Table S2.**

Summary of Hypothesis Testing

| Hypothesis | Proposed relationship | Result | Evidence/Comments |
| --- | --- | --- | --- |
| H1a (1) | PJD/PFD → Health-related work functioning (presenteeism) (−) | **Not supported** | Neither PJD nor PFD significantly predicted health-related work functioning (PJD: β = −.161, *p* = .262; PFD: β = −.124, *p* = .102). |
| H1a (2) | PJD/PFD → PSC (−) | **Not supported** | Neither PJD nor PFD significantly predicted PSC (PJD: β = −.045, *p* = .647; PFD: β = −.092, *p* = .077). |
| H1b (1) | SSTL → Health-related work functioning (presenteeism) (+) | **Not supported** | SSTL was not significantly associated with health-related work functioning (β = .048, *p* = .693). |
| H1b (2) | SSTL → PSC (+) | **Supported** | SSTL was positively and significantly associated with PSC (β = .493, *p* < .001). |
| H1b (3) | SSPL → Health-related work functioning (presenteeism) (−) | **Supported** | SSPL was significantly and negatively associated with health-related work functioning (β = −.514, *p* = .003), consistent with the hypothesised direction. |
| H1b (4) | SSPL → PSC (−) | **Supported** | SSPL was significantly and negatively associated with PSC (β = −.537, *p* = .004), consistent with the hypothesised direction. |
| H2a | PJD/PFD → Safety outcomes (−) | **Not supported** | Neither PJD nor PFD significantly predicted SC, SP, or SM. Moreover, several estimated coefficients were in the opposite direction to the hypothesised negative direction. |
| H2b (1) | SSTL → Safety outcomes (+) | **Not supported** | SSTL did not significantly predict SC (β = .036, *p* = .614), SP (β = .053, *p* = .514), or SM (β = .077, *p* = .575). |
| H2b (2) | SSPL → Safety outcomes (−) | **Not supported** | SSPL did not significantly predict SC (β = .155, *p* = .267), SP (β = .053, *p* = .321), or SM (β = −.086, *p* = .429). |
| H3a | Health-related work functioning (presenteeism) → Safety outcomes (+) | **Not supported** | Health-related work functioning was not significantly associated with SC (β = .015, *p* = .674), SP (β = −.020, *p* = .611), or SM (β = −.006, *p* = .838). |
| H3b | PSC → Safety outcomes (+) | **Partially supported** | PSC significantly predicted SP (β = .232, *p* < .001) and SM (β = .075, *p* = .048). The association with SC was at the conventional significance threshold (β = .093, *p* = .050) and was interpreted with caution in the manuscript. |
| H4a (1) | PJD/PFD → Health-related work functioning → Safety outcomes | **Not supported** | No significant indirect effects were identified for PJD or PFD through health-related work functioning. |
| H4a (2) | Leadership → Health-related work functioning → Safety outcomes | **Not supported** | Health-related work functioning did not significantly mediate the relationships between safety-specific leadership and the safety outcomes. |
| H4b (1) | PJD/PFD → PSC → Safety outcomes | **Not supported** | The indirect effects of PJD and PFD through PSC were not statistically significant. The manuscript explicitly reports H4b1 as unsupported. |
| H4b (2) | Leadership → PSC → Safety outcomes | **Supported** | SSTL had significant positive indirect effects through PSC on SC (β = .034, *p* = .045), SP (β = .080, *p* < .001), and SM (β = .025, *p* = .030). SSPL had significant negative indirect effects through PSC on SC (β = −.041, *p* = .033), SP (β = −.110, *p* = .001), and SM (β = −.030, *p* = .029). |
| H5a | Health-related work functioning moderates the relationship between JD and Safety outcomes | **Supported** | The interaction between job demands and health-related work functioning significantly predicted SC (β = −.122, *p* = .012). Simple-slope analyses indicated that better health-related work functioning buffered the negative association between job demands and safety compliance. |
| H5b | Health-related work functioning moderates the relationship between Leadership and Safety outcomes | **Not supported** | The interactions between health-related work functioning and safety leadership were not significant. |
| H6a | PSC moderates the relationship between Leadership and Safety outcomes | **Not supported** | No significant interactions involving PSC and safety leadership were observed. |
| H6b | PSC moderates the relationship between JD and Safety outcomes | **Not supported** | No significant interactions involving PSC and job demands were observed. |

**Note.** SC = Safety Compliance; SP = Safety Participation; SM = Safety Motivation; PJD = Perceived Job Demands; PFD = Perceived Family Demands; SSTL = Safety-Specific Transformational Leadership; SSPL = Safety-Specific Passive Leadership; PSC = Psychosocial Safety Climate. Supported indicates that all proposed relationships were statistically significant and consistent with the hypothesised direction. Partially supported indicates that support was observed for only a subset of the proposed pathways or safety outcomes. Not supported indicates that the proposed relationships were not statistically significant or were inconsistent with the hypothesised direction. Hypotheses H1a–H5 comprise the Health Impairment Pathway, whereas hypotheses H1b–H6 comprise the Motivational Pathway within the proposed JD–R–OHS framework.

**Table S3.**

Hypothesis Testing of All Indirect Effects and Mediating Pathways in the JD–R–OHS Model.

| **Effect** | ***β*** | ***p*** | **95% CI** | | **Direction** |
| --- | --- | --- | --- | --- | --- |
|  |  |  | **LL** | **UL** |  |
| PJD → Presenteeism → SP | .002 | .110 | -.084 | .009 | Positive |
| PJD → Presenteeism → SM | -.004 | .930 | -.055 | .006 | Negative |
| PJD → Presenteeism → SC | -.004 | .076 | -.068 | .010 | Negative |
| PFD → Presenteeism → SP | .004 | .060 | -.031 | .006 | Positive |
| PFD → Presenteeism → SM | -.006 | .073 | -.030 | .001 | Negative |
| PFD → Presenteeism → SC | -.006 | .080 | -.040 | .001 | Negative |
| SSTL → Presenteeism → SP | -.001 | .197 | -.014 | .051 | Negative |
| SSTL → Presenteeism → SM | .002 | .091 | -.010 | .031 | Positive |
| SSTL → Presenteeism → SC | .002 | .056 | -.017 | .043 | Positive |
| SSPL → Presenteeism → SP | .006 | .073 | -.041 | .009 | Positive |
| SSPL → Presenteeism → SM | -.009 | .198 | -.039 | .001 | Negative |
| SSPL → Presenteeism → SC | -.009 | .530 | -.085 | .000 | Negative |
| PJD → PSC → SP | -.006 | .860 | -.051 | .042 | Negative |
| PJD → PSC → SM | -.003 | .573 | -.037 | .019 | Negative |
| PJD → PSC → SC | -.004 | .614 | -.043 | .026 | Negative |
| PFD → PSC → SP | -.035 | .191 | -.082 | .016 | Negative |
| PFD → PSC → SM | -.016 | .078 | -.061 | .003 | Negative |
| PFD → PSC → SC | -.022 | .078 | -.069 | .004 | Negative |
| SSTL → PSC → SP | **.080** | **<.001** | **.040** | **.140** | **Positive** |
| SSTL → PSC → SM | **.025** | **.030** | **.006** | **.054** | **Positive** |
| SSTL → PSC → SC | **.034** | **.045** | **.010** | **.072** | **Positive** |
| SSPL → PSC → SP | **-.110** | **.001** | **-.228** | **-.049** | **Negative** |
| SSPL → PSC → SM | **-.030** | **.029** | **-.086** | **-.007** | **Negative** |
| SSPL → PSC → SC | **-.041** | **.033** | **-.114** | **-.010** | **Negative** |

*Note. PJD,* Perceived Job Demands; *PFD,* Perceived Family Demands; *SSTL,* Safety-Specific Transformational Leadership; *SSPL,* Safety-Specific Passive Leadership; *PSC,* Psychosocial Safety Climate; *SC,* Safety Compliance; *SP,* Safety Participation; *SM,* Safety Motivation.

**Table S4.**

Hypothesis testing of all direct and moderating effects in the moderating model of the JD-R-OHS framework.

| Predictor / Interaction | DV | *β* | *p* | 95% CI | |
| --- | --- | --- | --- | --- | --- |
| *Direct Effects* |  |  |  | **LL** | **UL** |
| Perceived Job Demands | SC | 0.048 | 0.376 | -0.071 | 0.165 |
|  | SP | 0.072 | 0.161 | -0.038 | 0.193 |
|  | SM | -0.031 | 0.576 | -0.146 | 0.081 |
| Perceived Family Demands | SC | 0.072 | 0.194 | -0.055 | 0.192 |
|  | **SP** | **0.160** | **0.003** | **0.066** | **0.253** |
|  | SM | 0.035 | 0.540 | -0.081 | 0.159 |
| Safety-Specific Transformational Leadership | SC | 0.090 | 0.230 | -0.070 | 0.240 |
|  | **SP** | **0.168** | **0.018** | **0.024** | **0.307** |
|  | SM | 0.006 | 0.942 | -0.171 | 0.181 |
| SSPL | SC | -0.063 | 0.339 | -0.198 | 0.076 |
|  | SP | 0.008 | 0.902 | -0.118 | 0.154 |
|  | SM | -0.095 | 0.161 | -0.259 | 0.056 |
| Presenteeism | SC | 0.101 | 0.066 | -0.019 | 0.218 |
|  | SP | 0.018 | 0.727 | -0.099 | 0.139 |
|  | **SM** | **0.170** | **0.002** | **0.073** | **0.269** |
| Psychosocial Safety Climate | **SC** | **0.193** | **0.004** | **0.074** | **0.312** |
|  | **SP** | **0.301** | **<.001** | **0.172** | **0.441** |
|  | **SM** | **0.148** | **0.029** | **0.025** | **0.257** |
| *Moderation Effects (Interactions)* |  |  |  |  |  |
| Perceived Job Demands × Presenteeism | **SC** | **-0.122** | **0.012** | **-0.214** | **-0.016** |
|  | SP | -0.076 | 0.100 | -0.202 | 0.036 |
|  | SM | -0.057 | 0.249 | -0.185 | 0.052 |
| Perceived Job Demands × Psychosocial Safety Climate | SC | -0.043 | 0.430 | -0.160 | 0.086 |
|  | SP | 0.018 | 0.727 | -0.095 | 0.145 |
|  | SM | -0.035 | 0.523 | -0.147 | 0.099 |
| Perceived Family Demands × Presenteeism | SC | 0.021 | 0.703 | -0.116 | 0.155 |
|  | SP | -0.042 | 0.418 | -0.172 | 0.086 |
|  | SM | 0.066 | 0.238 | -0.058 | 0.186 |
| Perceived Family Demands × Psychosocial Safety Climate | SC | -0.002 | 0.967 | -0.136 | 0.138 |
|  | SP | 0.053 | 0.301 | -0.082 | 0.176 |
|  | SM | -0.064 | 0.240 | -0.191 | 0.066 |
| SSTL × Presenteeism | SC | 0.080 | 0.244 | -0.074 | 0.236 |
|  | SP | 0.030 | 0.647 | -0.138 | 0.198 |
|  | SM | 0.003 | 0.967 | -0.174 | 0.188 |
| SSTL × Psychosocial Safety Climate | SC | 0.014 | 0.813 | -0.122 | 0.152 |
|  | SP | 0.017 | 0.768 | -0.146 | 0.169 |
|  | SM | 0.087 | 0.156 | -0.073 | 0.284 |
| SSPL × Presenteeism | SC | 0.076 | 0.257 | -0.072 | 0.232 |
|  | SP | -0.015 | 0.813 | -0.172 | 0.131 |
|  | SM | 0.003 | 0.966 | -0.159 | 0.180 |
| SSPL × Psychosocial Safety Climate | SC | -0.060 | 0.350 | -0.208 | 0.092 |
|  | SP | 0.004 | 0.944 | -0.163 | 0.162 |
|  | SM | -0.023 | 0.723 | -0.200 | 0.156 |

***Note.*** *DV,* Dependent Variable; *SSTL,* Safety-Specific Transformational Leadership; SSPL, Safety-Specific Passive Leadership; *SC,* Safety Compliance; *SP,* Safety Participation; *SM,* Safety Motivation. β values and p values are from the maximum-likelihood regression weights; 95% CIs are the bias-corrected percentile bootstrap intervals from the AMOS output. Statistically significant effects (p < .05) are bolded.
